# A database of chemical absorption in human skin with mechanistic modeling applications

**DOI:** 10.1038/s41597-024-03588-3

**Published:** 2024-07-10

**Authors:** Jessica N. Stevens, Alyson K. Prockter, Hunter A. Fisher, Hien Tran, Marina V. Evans

**Affiliations:** 1 Department of Mathematics, North Carolina State University, Raleigh, NC USA; 2 Oak Ridge Associated Universities (ORAU) assigned to United States Environmental Protection Agency (USEPA), Office of Research and Development (ORD), Research Triangle Park, NC USA; 3 United States Environmental Protection Agency (USEPA), Center for Computational Toxicity and Exposure, Office of Research and Development (ORD), Research Triangle Park, NC USA

**Keywords:** Chemical libraries, Data publication and archiving

## Abstract

Whether from environmental and occupational hazards or from topical pharmaceuticals, the human skin comes into contact with various chemicals every day. *In vivo* experiments not only require large investments of both time and money, but *in vivo* experiments can also be unethical due to the need to intentionally or incidentally expose humans or animals to toxic chemicals. Comparatively, *in vitro* experiments offer ethical and financial advantages when combined with the opportunity to selectively choose chemicals for experimentation. With *in vivo* experimentation being so infeasible, many scientists have chosen to make their *in vitro* data available publicly. Using these data, a detailed database containing 73 chemicals was created with a robust set of descriptors to be used in connection with mathematical modeling to predict diffusion, permeability, and partition coefficients. This resulting database is tailored to be easily used in various coding languages.

## Background & Summary

Skin is the largest organ in the human body and functions primarily to protect the body from external factors. Due to the key role skin plays in safeguarding the body, understanding how chemicals penetrate has applications across multiple disciplines. Most notably, chemical penetration of human skin has significance with regard to determining the risk and toxicity of environmental and occupational hazards as well as the efficacy of topical pharmaceuticals. With the knowledge of skin absorption growing in importance for various fields, the need for a database comprised of chemicals and their dermal absorption parameters, such as permeability and diffusion coefficients also grows. *In vivo* experimentation often requires large investments of time and money and may involve ethical issues; for this reason *In vivo* experimentation is not always plausible. *In silico* tools require toxicokinetic datasets to be able to simulate a wide variety of chemicals. “High throughput toxicokinetics”, or httk^1^, and other models will benefit from having open access datasets with physiochemical parameters and diffusion descriptors. Total accumulation over time can be used to quantify dermal absorption parameters, such as permeability and as such, multi-linear regression techniques have also been applied to dermal permeability datasets to obtain QSAR (Quantitative Structural Activity Relationships) equations for different exposure scenarios^2–4^.

Earlier dermal absorption *in vitro* experiments separated the upper layers of skin (largely referred to as the whole epidermis) to quantify dermal absorption parameters. The assumption that the uppermost skin layers offered the highest resistance to absorption motivated the experimental choice for epidermis use^5^. The number of layers included in the *in vitro* experiments has varied over time. Since the highest barrier to dermal penetration has been thought to be in the upper layers (the stratum corneum and the viable epidermis), earlier *in vitro* experiments included only these layers. However, inclusion of a partial dermis has become common practice when using the data from dermatomed experiments. Unlike the epidermis, which includes a lipid barrier in the stratum corneum (typically modeled by a “brick and mortar” structure^6,7^), the dermis is an aqueous barrier that contains collagen and plasma proteins contributing to binding, and facilitates capillary transport^8^ For this work, data was compiled across the epidermis, stratum corneum, and dermis in order to create a unique and accessible database.

An earlier skin database (HuskinDB) has been published in *Scientific Data*, but it is limited to inclusion of permeability coefficients only^3^. Our work added diffusion and partition coefficients for each layer. The permeability coefficients (*k*_*p*_) can be related to the diffusion coefficients (*D*) and partition coefficients (*P*) using the ideal membrane equation when the layer depth (*l*) is known^9^: ${k}_{p}=\frac{P\ast D}{l}$. Further, our database also includes chemical descriptors for each identified chemical, adding the ability to explore QSAR models such as the Potts-Guy model.

Our created database contains publicly available experimental data that were collected from multiple sources. Experimental permeability and diffusion coefficient values^10–12^ along with chemical descriptors^13–15^ were collected and included in the database. In addition to compiling diffusion and permeability coefficients across three layers, another valuable aspect of this database is the focus on chemical features which includes those that are indicative of volatility such as melting point and vapor pressure. Volatility was not explored in the Potts-Guy Equation^16^, which is often cited when discussing skin permeability. Including these features is unique, as volatility is largely unexplored in regards to dermal absorption. A major application of the database is to use it in connection with mathematical modeling to quantify and predict permeability, partition, and diffusion coefficients.

## Methods

The data were compiled from the literature and began with 50 cosmetic chemicals from one source that were measured for penetration in the skin under a standardized protocol in aqueous buffers^10^. The database was further expanded to include non-volatile chemicals^11^ and hydrocortisones^12^ for a total of 73 distinct chemicals that are identifiable by name, CAS (Chemical Abstracts Service) number, DSSTox (Distributed Structure-Searchable Toxicity) Substance ID, and SMILES (Simplified Molecular Input Line Entry System).

### Source identification

Identification of a potential data source from the open literature was a key step for the development of this database. PubMed and GoogleScholar were used as primary search engines. Query phrases used included “human dermal absorption”, “aqueous vehicles”, “*in vitro* measurements”, and “epidermis, SC, and dermis”.

The search was limited to publication between the years 1970 and 2022; details for the experimentation leading to data collection was required to be provided within the publication itself. Once a manuscript was identified, a researcher read the paper and decided if the data reported met the selection criteria. The criteria specified were: human skin, *in vitro* experiments, aqueous vehicle, and known dose. The researcher determined if the data published could be used for the database. The publications included in this database evaluated drug permeation utilizing Franz diffusion cells with human skin plugs that were removed during surgery. This, however, is a criteria that was not determined a priori.

Recent publications typically include a table or electronic dataset reporting the values. Older manuscripts had their data entered by hand and curated by two separate individuals followed by a verification by a third. In all cases, the data were regarded as valid as reported. Only unit conversions were performed by the researcher to ensure that all data in this database had consistent units.

### Data content

The following criteria were considered prior to including data from a publication:

- The publication was publicly accessible
- The primary source of data were the publication or the associated excel file
- The units were included or able to be determined from the publication’s text
- The permeability coefficient (*k*_*p*_) and/or diffusion coefficient was included with specifications of the layer(s) or could be calculated from other data
- Any chemical vehicle(s), in addition to the aqueous buffer, were identified.

As as result of this criteria, the three sources of permeability and diffusion coefficients used in this database are Ellison *et al*.^10^, Krestos *et al*.^11^, and Anderson *et al*.^12^. Experimentation is detailed in the corresponding publications and was reviewed by all researchers to ensure all necessary criteria was met.

In order to provide a consistent set of chemical descriptors, features not included with the experimental data were pulled from the EPA’s CompTox Chemicals Dashboard^13^ as well as the PaDEL-Descriptor^15^ and PubMed^14^ to allow for a more robust set of features, including structural information as well as the highlighted features below:

- Molecular Weight (*MW*)
- Vapor Pressure
- Index of Refraction
- Molar Refractivity
- Henry’s Constant
- Polarizability
- Surface Tension
- Molar Volume
- Boiling Point
- Melting Point
- Water Saturation (*S*_*w*_)
- Octanol-Water Partition Coefficient ($\log P$)
- Bioconcentration Factor
- Biodegradation Half Life
- Michaelis constant (*K*_*m*_)
- Atmospheric Hydroxylation Rate
- Water Solubility
- Density
- Flash Point
- Soil Adsorption Coefficient

Some features were reported more than once in the event of a unit conversion such as *k*_*p*_, which is reported in both centimeters/hour and centimeters/second. In the event that the data were unavailable for a specific chemical, the entry was left blank and that chemical was not included in any analysis of that feature. A list of features and the corresponding units, excluding some features that are dimensionless, can be found in Tables 1–4.

**Table 1:** Units and approximate ranges of various features.

Feature Name	Units	Range of Values
Molecular Weight (*MW*)	g/mol	18.015 to 518.647
Vapor Pressure (*P*_*v*_)	mmHg	2.12 × 10^−15^ to 760
Octanol-Water Partition Coefficient ($\log P$)		−2.824 to 4.946
Octanol-Air Partition Coefficient ($\log {k}_{OA}$)		2.189 to 11.107
Diffusion Coefficient (*D*)	cm^2^/s and cm^2^/h	varies per layer, see Tables 2–4
Permeability Coefficient (*logk*_*p*_)	cm/s and cm/h	varies per layer, see Tables 2–4
Melting Point	°C	−126.1 to 237.711
Boiling Point	°C	64.7 to 685.5
Density	g/cm^3^	0.789 to 1.6
Molar Refractivity	f^3^	20.8 to 123.74
Molar Volume	m^3^/mol	70.2 to 382.4
Water Solubility	mg/L	1.217 × 10^−5^ to 73
Water Saturation (*S*_*w*_)	mg/cm^3^ at 32°C	0.027 to 531
Surface Tension	N/m	20.14 to 92.1
Polarizability	10^−24^ cm^3^	8.2 to 49
Flash Point	°C	9.7 to 316
Topological Surface Area	Å/molecule	0 to 144
Biodegradation Half Life	days	0.01 to 97.932
Michaelis constant (*K*_*m*_)		0.0663 to 4.246
Extent of Linearity	hours	varies per layer, see Tables 2–4
Experimental Lag Time	hours	varies per layer, see Tables 2–4

**Table 2:** Units and approximate ranges of various features in the epidermis.

Feature Name	Units	Range of Values
Diffusion Coefficient (*D*)	cm^2^/s	6.684 × 10^−12^ to 1.318 × 10^−6^
Permeability Coefficient (*logk*_*p*_)	cm/s	− 8.14 to − 3.78
Extent of Linearity	hours	3.931 to 22
Experimental Lag Time	hours	0.382 to 6.434

**Table 3:** Units and approximate ranges of various features in the stratum corneum.

Feature Name	Units	Range of Values
Diffusion Coefficient (*D*)	cm^2^/s	0 to 2.268 × 10^−7^
Permeability Coefficient (*logk*_*p*_)	cm/s	− 8.141 to − 3.849
Extent of Linearity	hours	4.08 to 22
Experimental Lag Time	hours	0.353 to 7.551

**Table 4:** Units and approximate ranges of various features in the dermis.

Feature Name	Units	Range of Values
Diffusion Coefficient (*D*)	cm^2^/s	4.444 × 10^−11^ to 6.303 × 10^−5^
Permeability Coefficient (*logk*_*p*_)	cm/s	− 5.955 to − 2.399
Extent of Linearity	hours	4.182 to 18.222
Experimental Lag Time	hours	0.707 to 5.490

### Data usage and calculations

**Fig. 1:**
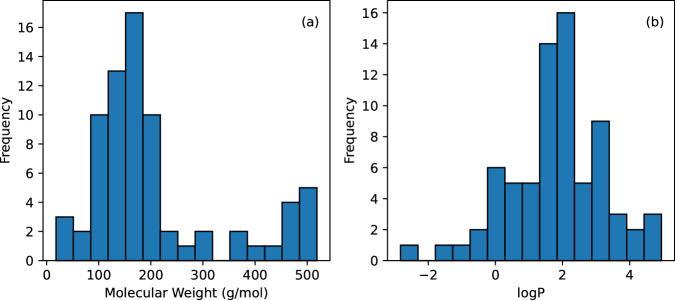
(**a**) Distribution of molecular weight of all chemicals, (**b**) distribution of $\log P$ values for all chemicals.

**Fig. 2:**
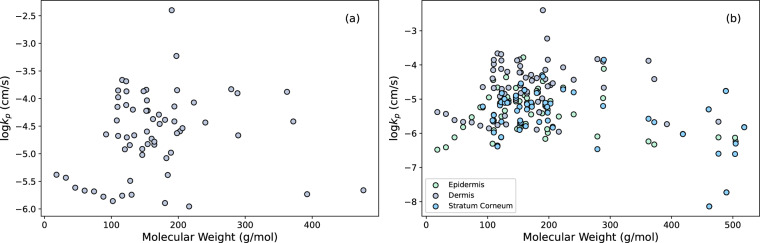
Molecular weight versus the value of $\log {k}_{p}$ of all chemicals in (**a**) the dermis and (**b**) all layers.

**Fig. 3:**
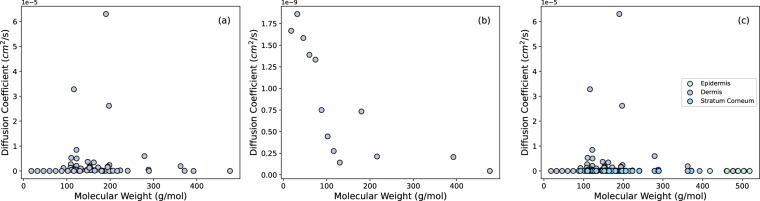
Molecular weight versus the diffusion coefficients for (**a**) all chemicals in the dermis (**b**) non-volatile chemicals in the dermis, (**c**) all chemicals in all layers.

Figure 1 shows the distributions for molecular weight (Fig. 1a) and $\log P$ (Fig. 1b). The values for molecular weights for the chemicals fall between 18 g/mol and 519 g/mol whereas the values for $\log P$ fall between -3 and 5. In Fig. 2, molecular weight is plotted against the values for $\log {k}_{p}$ in the dermis (Fig. 2a) and all layers of the skin (Fig. 2b). While certain subsets of the data may show a trend, the data overall do not indicate a correlation between $\log {k}_{p}$ and molecular weight. Similarly in Fig. 3, the diffusion coefficients are plotted against the molecular weights.

The relationship between the diffusion coefficients (cm^2^/s) in the dermis and molecular weight (g/mol) for non-volatile chemicals shown in Fig. 3b indicates a negative correlation between the two. It is important to note, however, that the figure only includes a small subset of the chemicals in a single layer of skin. The other plots in Fig. 3, and the data in this compiled database, show no significant correlation between the molecular weights and diffusion coefficients despite the common assumption that larger chemicals would have lower diffusion coefficients. This lack of correlation further supports the need for a robust database with various features that may contribute to QSAR models in varying degrees.

**Fig. 4:**
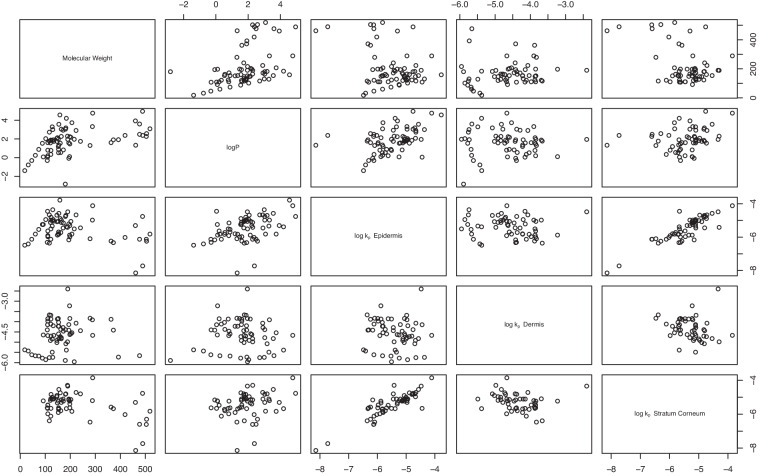
Scatter plot matrices for molecular weight, $\log P$, and $\log {k}_{p}$ values in all layers.

Dermal permeability (*k*_*p*_) is probably the most common parameter used to estimate dermal penetration and net absorption. Using ideal membrane theory, the diffusion constant is directly proportional to permeability, although modified by partitioning and membrane depth. The Potts-Guy correlation equation^16^ describe a direct relationship between $\log {k}_{p}$ and $\log P$, particularly for the epidermis (consisting of the stratum corneum and viable epidermis) skin barrier. Figure 4 summarizes individual correlations between $\log {k}_{p}$, *MW*, and $\log P$ for different layers.

The main aim of this dermal database was to aid in the development of mathematical models and computer simulations such that more information can be learned and extrapolated regarding how chemicals diffuse and permeate within the skin’s layers. The example chosen for this paper is the mechanistic modeling of dermis diffusion coefficient since the diffusion constant is rarely included in QSAR models.

### Example applications for dermis layer

**Fig. 5:**
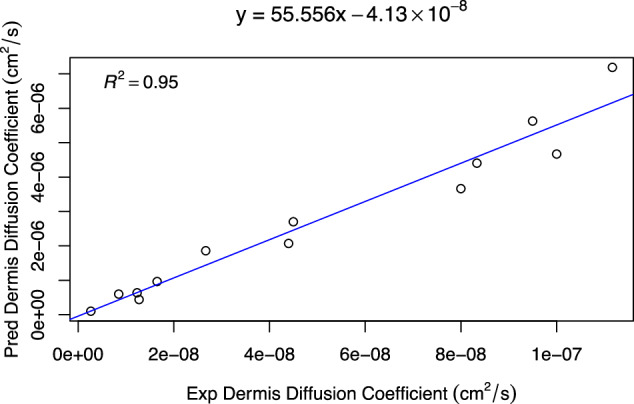
Predicted diffusion coefficient vs experimental values collected in dermis. Chemical descriptors used: *MW*, $\log P$, and *f*_*u*_ (fraction unbound).

Since the dermis contains plasma proteins, an additional descriptor included was fraction unbound in the plasma (*f*_*u*_). The dermis diffusion constant was calculated using the diffusion equation presented by Chen *et al*.^17^. Predictions were compared to experimental values obtained from Hewitt *et al*. and Kretsos *et al*.^10,11^ Fig. 5 presents the results for the Kretsos dataset containing the 13 chemicals.

## Data Records

The database is deposited on the Dryad Digital Repository as a series of Microsoft Excel files prepared to be used in coding^18^. It is presented as individual files for each layer (epidermis, stratum corneum, dermis) and chemical type (fragrance related, non-volatile, hydrocortisone). Additional spreadsheets containing all information, the chemical descriptors, and time course data are also included along with a notated and color-coded file which is condensed and not recommended for coding.

## Data Validation

The collection of experimental data was collected from its corresponding publication^10–12^ and the additional features were collected from the EPA CompTox Chemicals Dashboard (version 2.2.0)^13^, Padel-descriptor^15^, as well as additional literature^14,19^. The database was curated by a team of two and reviewed by an additional team member in order to ensure that the data were accurately reported with correct units. The dermal absorption coefficients were collected from peer-reviewed publications and included in the database, taking into account any additional supplementary materials and corrections.

## Technical Validation

Chemicals identifiers were used as reported in the open literature. Many publications used CAS numbers to identify the chemical. If a chemical name was given without CAS number, the US EPA Dashboard was used to obtain unique identifiers for each chemical (CAS number and DSSTox ID). The Dashboard has a synonym function designed specifically to identify chemicals by different names. PubChem was also used to further confirm a chemical’s identity and corresponding DSSTox ID. Agreement between these different sources ensured that the chemicals were correctly identified and are provided for the users’ convenience.

To obtain the fraction unbound needed for the dermis calculations, OPERA (version 2.8)^20^ was used. The fraction unbound predictions from OPERA are also included in the EPA CompTox Chemicals Dashboard. However, extracting multiple values for different chemicals is easily done in the original software. A function within the US EPA HTTK package can also be used to download multiple values for fraction unbound if desired. The fraction unbound was only needed in the dermis, since this layer contains plasma proteins that exhibit binding and affect overall absorption into the dermis. Because the dermis is important for capillary absorption into the blood stream, the additional descriptors were a valuable addition.

## Usage Notes

The database is built in order to be easily integrated into coding, particularly with R Studio^21^. The spreadsheet is formatted such that it can be used as a whole and simultaneously functions as separated databases for each layer and subset of chemicals.

## Data Availability

The database as well as the code and separated data for the predicted values presented in Fig. 5 are available on Dryad^18^.
